# A Fusion Strategy for Vehicle Positioning at Intersections Utilizing UWB and Onboard Sensors

**DOI:** 10.3390/s24020476

**Published:** 2024-01-12

**Authors:** Huaikun Gao, Xu Li, Xiang Song

**Affiliations:** 1 School of Instrument Science and Engineering, Southeast University, Nanjing 210096, China; tx2ghk@163.com; 2 School of Electronic Engineering, Nanjing Xiaozhuang University, Nanjing 211171, China; sx2190105@163.com

**Keywords:** AIMM, ARIMA–GARCH, NLOS, onboard sensors, urban scenarios, UWB, vehicle positioning

## Abstract

For vehicle positioning applications in Intelligent Transportation Systems (ITS), lane-level or even more precise localization is desired in some typical urban scenarios. With the rapid development of wireless positioning technologies, ultrawide bandwidth (UWB) has stood out and become a prominent approach for high-precision positioning. However, in traffic scenarios, the UWB-based positioning method may deteriorate because of not-line-of-sight (NLOS) propagation, multipath effect and other external interference. To overcome these problems, in this paper, a fusion strategy utilizing UWB and onboard sensors is developed to achieve reliable and precise vehicle positioning. It is a two-step approach, which includes the preprocessing of UWB raw measurements and the global estimation of vehicle position. Firstly, an ARIMA–GARCH model to address the NLOS problem of UWB at vehicular traffic scenarios is developed, and then the NLOS of UWB can be detected and corrected efficiently. Further, an adaptive IMM algorithm is developed to realize global fusion. Compared with traditional IMM, the proposed AIMM is capable of adjusting the model probabilities to make them better matching for current driving conditions, then positioning accuracy can be improved. Finally, the method is validated through experiments. Field test results verify the effectiveness and feasibility of the proposed strategy.

## 1. Introduction

With the booming development of traffic and transportation in recent years, there is a rapidly growing demand for reliable and precise positioning for land vehicles. Meter-level or even lane-level localization is desperately required in many vehicles’ safety and guidance-related applications such as Advanced Driver Assistance Systems (ADAS), Intelligent Transportation Systems (ITS), and autonomous driving [1,2], etc.

A widely spread method for vehicle localization is the Global Navigation Satellite System (GNSS), which is capable of providing position, velocity, and time information globally as long as the user has an unobstructed view of the sky [3]. However, in GNSS-challenging environments such as urban canyons, flyovers or intersections with many high-rise buildings around, satellites’ signal blocking by surrounding buildings may seriously decrease the positioning accuracy. If there are less than four satellites in sight, the GNSS even fails to work [4,5,6].

Hence, to provide reliable, accurate, and continuous positions in urban scenarios, the positioning system should be augmented with additional sensors such as inertial measurement unit (IMU), in-vehicle sensors, digital maps, cameras, radar, or laser scanners [7].

Due to system complementary characteristics, the most common method is that whereby the GNSS is augmented with IMU [8]. In [9,10], continuous and integrated positioning is achieved through GNSS/INS fusion during short-time GNSS outages. Georgy J. et al. introduced an integration of an odometer with GNSS and gyro in [11]. However, in view of expenditures, most land vehicle applications can only afford low-cost onboard sensors based on microelectromechanical system (MEMS) technology [12]. During long-time GNSS outage scenarios such as tunnels or urban canyons, the accuracy of those methods deteriorated rapidly over time because of sensor error accumulation. Sensors fusion between GNSS and vision perform quite well under certain circumstances [13,14,15], but those methods degrade in some cases such as unstructured roads or poor light environments. Similarly, LiDAR-aided solutions [16,17] are vulnerable to disruptions under adverse weather conditions.

For many location applications in ITS, lane-level or even more precise localization is desired in some typical urban scenarios such as urban intersections, where those supplementary methods mentioned above may be deficient. Thus, wireless positioning technologies such as a Wireless Local Area Network (WLAN), Radio Frequency Identification (RFID), and Ultra-Wide Bandwidth (UWB) are also investigated in [18,19,20]. Compared with other wireless technologies such as RFID, UWB offers several benefits, including accurate ranging and robustness to jamming and interference [21,22]. Although UWB is mainly used for indoor positioning, it is recognized as a feasible assistant approach for outdoor applications in dense urban areas [23,24]. Due to their excellent capability of penetrating obstacles and resolving the multipath aspect, those methods utilizing UWB stand out and show great potential for achieving high ranging accuracy [25]. 

Generally, in a vehicle-to-infrastructure (V2I) architecture utilizing UWB, roadside nodes will transmit their coordinates to surrounding vehicles as well as radio signal measurements. The location of vehicles is estimated by combining radio signal measurements from roadside nodes. However, radio signal propagation is easily blocked by surrounding buildings, trees, or passing-by vehicles, the wireless positioning method may suffer from not-line-of-sight (NLOS) propagation, multipath effect, and multi-user interference. Despite UWB having the capacity of penetration as well as immunity of multipath, NLOS propagation still occurs frequently in intricate environments, which may lead to a deterioration of positioning accuracy [26]. Therefore, many research studies [27,28,29] have been conducted to focus on NLOS detection and mitigation for UWB in dense environments. Whereas those methods are mainly applied in indoor localization, their performance has seldom been validated outside. NLOS detection and mitigation in vehicular traffic scenarios is exigently worth researching before UWB can be deployed [30]. In this paper, a novel approach based on nonlinear time series analysis, namely the autoregressive integrated moving average–generalized autoregressive conditionally heteroscedastic (ARIMA–GARCH) model, is introduced to cope with the rough errors in NLOS measurements.

Besides augmenting vehicle positioning systems with the aforementioned supplementary sensors, another crucial aspect of improving positioning performance is the fusion algorithm. To fuse information from multiple sensors, numerous Bayesian filter-based fusion algorithms, such as the Extended Kalman Filter (EKF), Unscented Kalman Filter (UKF), and Particle Filter (PF) [31,32,33], have also been proposed and refined over time. According to [34], the choice of an adequate process model is crucial for positioning performance. In most of the Bayesian filter-based methods, only one single process model, whether simple or complicated, is built and utilized. However, in reality, it seems difficult to choose an optimal model to capture all driving conditions.

Alternatively, the Interacting Multiple Model (IMM) approach was proposed to overcome this problem. It has drawn considerable attention recently, as it provides a valuable concept for maneuvering target tracking as well as ego-positioning [35,36]. In those research studies, the IMM filters contributed to adapting different maneuvering or driving conditions by model interacting and then provided a combined solution for vehicle behavior. In traditional IMM methods, the transition probabilities of each model are derived from the incorporated measurements and the estimation itself; those approaches make the IMM completely self-contained [37], which means that no additional information is necessary for the estimation. However, there are many situations in which additional knowledge is available [38].

To achieve reliable and accurate localization at typical urban intersections, this paper proposes a multisensor fusion strategy utilizing UWB and onboard sensors. This strategy adopts a two-step approach: the algorithms for both UWB range measurements preprocessing and global fusion are developed to realize better performance. The most significant and innovative parts of this paper are summarized as follows:

(1) An ARIMA–GARCH model to address the NLOS problem of UWB-aided positioning in vehicular traffic scenarios is proposed. Compared with the ARIMA model used in our previous work [30], this model can capture prominent characteristics of UWB measurements, such as self-similarity in the large time scale and multifractal in small time scale, and then the coarse error in range measurements of NLOS anchors could be mitigated more efficiently. With NLOS detection and mitigation of UWB raw information, the performance of subsequent global fusion can be significantly improved.
(2) An adaptive IMM algorithm is proposed to conduct global fusion, in which the low-cost onboard sensors, including wheel speed sensors, an electronic compass, and MEMS-INS, are utilized to be fused with UWB. The adaptive algorithm based on Bayesian inference, which incorporates additional UWB and steering sensor information into the multiple-model estimation process, can model the transition probabilities of the Markovian matrix adaptively. Compared with traditional IMM methods, the proposed AIMM model will adjust the model probabilities to make them better matching for current driving conditions, thus leading to better positioning performance.

This article is arranged as follows. Section 2 describes the outline of the proposed positioning strategy aided by UWB. The NLOS preprocessing algorithm is discussed in Section 3. Section 4 presents the AIMM fusion strategy. Field experimental results are displayed in Section 5. The concluding remarks are presented in Section 6.

## 2. Outline of The Proposed Multi-Sensor Fusion Strategy

**Figure 1:**
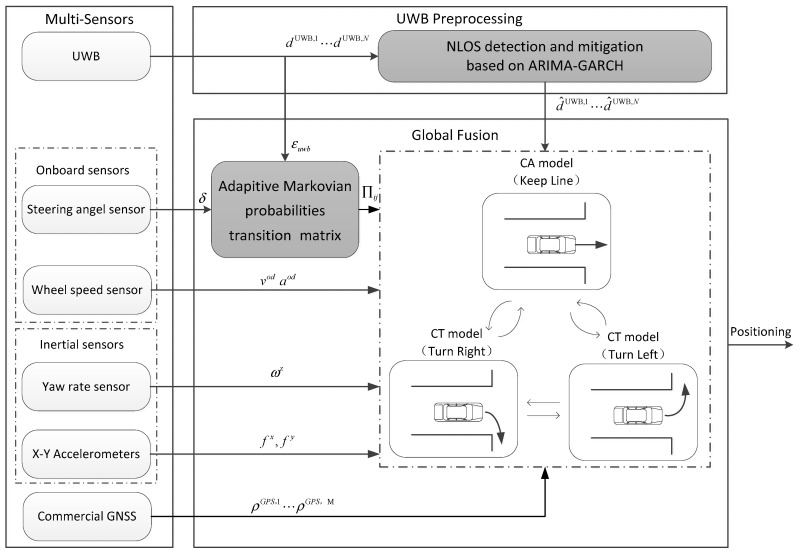
Proposed multi-sensor fusion strategy for vehicle positioning at intersections.

The proposed multi-sensor fusion strategy is made up of three components: the multi-sensor module, the UWB data preprocessing module using ARIMA–GARCH, and the global fusion module utilizing AIMM. The outline of the strategy is shown in Figure 1.

The multi-sensor module contains wheel speed sensors, a steering angle sensor, an electronic compass, and commercial-grade GNSS and MEMS inertial sensors. Note that, in this paper, only two orthogonal accelerometers along the longitudinal and lateral axes of the vehicle body frame and a vertical yaw gyro are utilized.

The ARIMA–GARCH model is developed for NLOS detection and mitigation. The range acquired through the time of arrival (TOA) measurement of UWB is apparently biased when there is a sudden transition from LOS to NLOS in vehicle traffic scenarios. In view of this, time series analysis can be used for anomaly detection. The most common and effective approach used for modeling time series is ARIMA. However, the ARIMA is a linear time series model which assumes constant variance. Yet in reality, UWB measurement series have shown the existence of conditional variance, especially when the NLOS situation occurs frequently. If such heteroscedasticity cannot be captured, the least-squares algorithm used to estimate the parameters of ARIMA will be biased [39]. Hence, an ARIMA–GARCH model is constructed and used to capture prominent characteristics of UWB measurements, such as self-similarity in the large time scale and multifractal in the small time scale, and then the abnormal error in range measurements of NLOS anchors can be identified and mitigated effectively.

The adaptive IMM algorithm is conducted to fulfill the global fusion. The constant turn (CT) and constant acceleration (CA) models are employed to capture two typical vehicle movements, that is, curvilinear and rectilinear motions, respectively. In the fulfillment of IMM filtering, an adaptive algorithm based on Bayesian inference, which incorporates additional UWB information into the multiple-model estimation process, is developed to model the transition probabilities of the Markovian matrix adaptively. By using the established AIMM model, the probabilities of the curvilinear and rectilinear motion models are more proper for actual driving conditions, and thus a better estimation of the vehicle position is achieved at urban intersections.

## 3. NLOS Mitigation Algorithm of UWB Anchors

There are four common methods for wireless positioning, namely receive signal strength indication (RSSI), angle of arrival (AOA), time of arrival (TOA), and time difference of arrival (TDOA). In this paper, UWB range measurements are obtained by the TOA method.

### 3.1. Outline of UWB Positioning

The benefits of exploiting UWB to realize vehicle positioning at intersections are as follows:

(a) UWB has an extremely large bandwidth, usually between the range of 3.6 GHz~10.1 GHz. In urban traffic environments, large bandwidth makes UWB robust against external interference;
(b) It can penetrate through some obstacles due to its impulse radio (IR) signal;
(c) Through the TOA method, UWB can achieve high ranging accuracy at the decimeter or even centimeter level, which may enhance positioning performance greatly.
(d) Besides, UWB is recognized as one potential technology for short distance high speed wireless communication, which is suitable to most vehicle-vehicle and vehicle-infrastructure applications in ITS.

**Figure 2:**
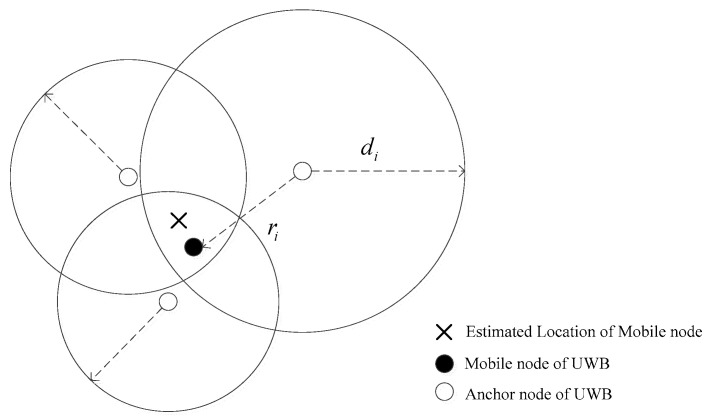
Illustration of UWB positioning based on ranging.

As shown in Figure 2, there is a UWB positioning system where several fixed UWB anchor nodes are deployed (the location of each anchor node can be acquired before they are deployed). Obviously, the range measurements are not equal to the true distances for the sake of several factors, such as not-line-of-sight propagation, multipath propagation, and measurement noise, etc. $d_{i}$ is the measured range between the mobile node and anchors modeled as:

(1) $$d_{i}=c\cdot (t_{prop}/2)=r_{i}\;+\;n_{i}\;+\;m_{i}\;+\;b_{i}\;i=1,2,\ldots ,N$$

where $t_{i,prop}$ is the round-trip flight time between the mobile node and the ith anchor, c is the speed of light, and $r_{i}$ is the real distance between the ith anchor and the mobile node. The measurement noise $n_{i}$ is usually modeled as additive white Gaussian noise ( $n_{i}\sim N(0,\sigma_{i}^{2})$ ).

The error term $m_{i}$ is caused by multipath propagation. In enclosed spaces, $m_{i}$ can be a nonnegligible component of range bias. However, when comparing with indoor environments, the chances of multipath propagation are low in open areas such as intersections. Owing to UWB’s excellent ability to resolve multipath, the multipath error term $m_{i}$ is assumed to be approximately equal to zero in this paper.

**Figure 3:**
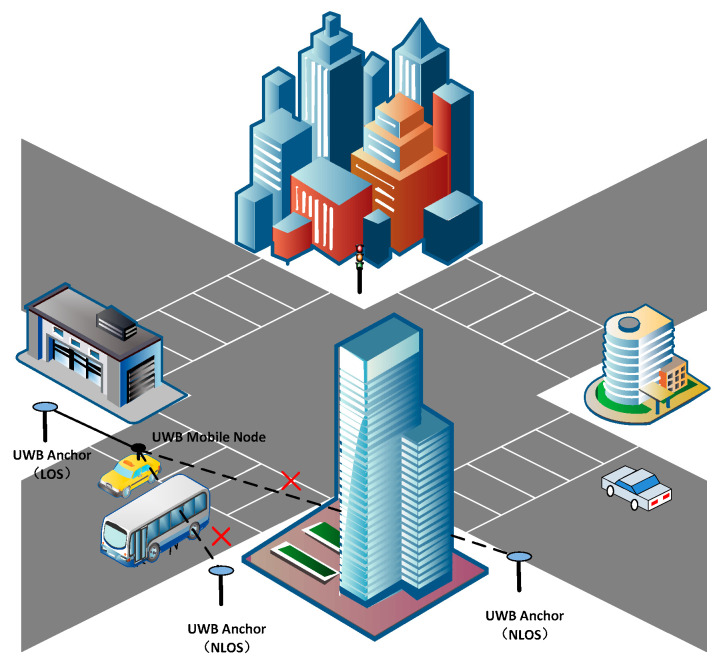
Typical NLOS situation at urban intersection scenarios.

Radio signal propagation between the UWB mobile node and anchors may be obstructed by surrounding buildings, trees, or passing-by vehicles at urban intersections. The typical NLOS situations are shown in Figure 3.

The positive range error $b_{i}$ is caused by blocking of the direct path.

(2) $$b_{i}=\left\{\begin{array}{ll} 0 & if\;ith\;anchor\;is\;LOS\\ \delta_{i} & if\;ith\;anchor\;is\;NLOS\; \end{array}\right.$$

### 3.2. NLOS Detection and Mitigation Algorithm

Compared with LOS propagation, the bias item $b_{i}$ is apparently larger in NLOS situations and leads to positioning accuracy deterioration. In view of this, a novel approach based on time series analysis is introduced to detect and mitigate the NLOS error of UWB anchors. In our application, the time series of UWB range measurements are definitely nonstationary.

The autoregressive integrated moving average model (ARIMA), which is brought forward by Box and Jenkins, is one of the most common and useful methods to model and analyze nonstationary time series. Generally speaking, ARIMA (p, d, q) consists of AR (p), MA (q), and ARMA (p, q) models [27].

For the time series of UWB range measurements d(k) (k=1,2,…,n) , suppose that the ARIMA (p, d, q) model can be expressed as:

(3) $$\begin{array}{ll} \hat{d}(k)= & a_{1}d(k-1)+a_{2}d(k-2)+\ldots +a_{p}d(k-p)+\\ & \varepsilon (k)-b_{1}\varepsilon (k-1)-b_{2}\varepsilon (k-2)-\ldots -b_{q}\varepsilon (k-q) \end{array}$$

where $a_{1},a_{2},\ldots ,a_{p}$ are parameters of the autoregressive component and $b_{1},b_{2},\ldots ,b_{q}$ are the moving average parameters. $\hat{d}(k)$ is the predication of d(k) .

Note that the ARIMA model proposed above is a linear time series model which assumes constant variance of ε(k),ε(k−1),…,ε(k−q) ; Yet in reality, UWB measurement series have shown the existence of conditional variance, especially when an NLOS situation occurs frequently. If such heteroscedasticity cannot be captured, the calculations of the ARIMA model parameters will be biased.

One approach to addressing this issue is to model the heteroscedasticity as a nonlinear association between consecutive errors. The GARCH model [40], which has time-varying variance, can help to better characterize the fluctuation features of UWB range series.

Once there is a significant heteroscedasticity of the residual series ε(k) generated by the ARIMA model, the GARCH model can be applied for capturing the heteroscedasticity. The GARCH (p,q) model is defined as:

(4) ε(k)=e(k)⋅h(k)

(5) $$h^{2}(k)=\alpha_{0}+\sum_{i=1}^{p}\alpha_{i}\varepsilon^{2}(k-i)+\sum_{j=1}^{q}\beta_{j}h^{2}(k-j)$$

where ε(k) is the residual at time *k* generated by the ARIMA model, e(k) ~N(0,1), $h^{2}(k)$ is the conditional variance at time *k*. $\alpha_{i}$ and $\beta_{j}$ are nonnegative constants.

**Figure 4:**
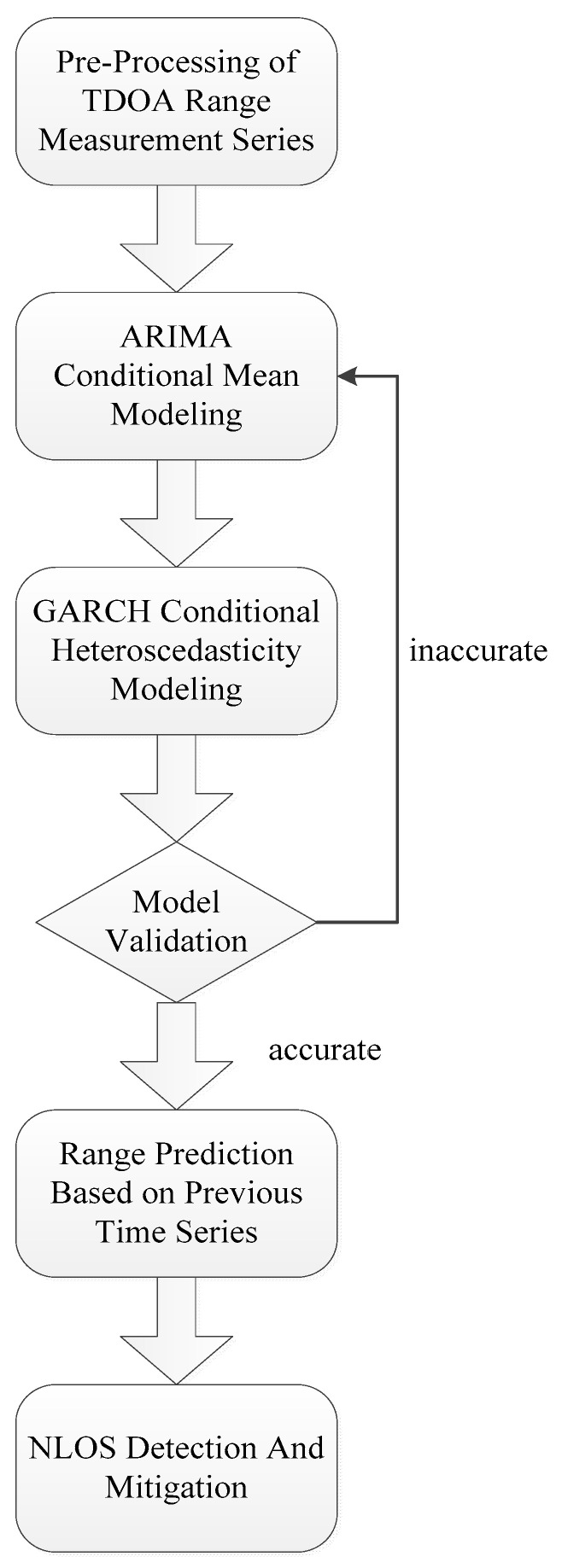
The procedure of fitting the ARIMA–GARCH model for NLOS mitigation.

As shown in Figure 4, the following steps are taken to construct the ARIMA–GARCH model for NLOS detection and mitigation:

(Step 1): Preprocessing the measured TOA range series to obtain a nearly stationary series. In this paper, we utilize the augmented Dickey–Fuller (ADF) test to determine whether or not the processed time series is stationary.

(Step 2): Fitting the ARIMA model with the UWB range series. This includes determining the order of p, q, d and estimating the parameters $a_{1},a_{2},\ldots ,a_{p}$ and $b_{1},b_{2},\ldots ,b_{q}$ . First-order differencing of the residual series is adequate in general (namely d = 1). Generally, the autocorrelation function (ACF) and its partial autocorrelation function (PACF) are applied to identify the order of p and q. The ACF measures the correlation between a variable and its past values, with ‘n’ representing the number of lags or time periods between these values. On the other hand, the PACF measures the correlation between a variable and its past values, but only with respect to a different variable. This helps in determining the number of lags in an AR model. By analyzing both the ACF and PACF plots, we can estimate the values of p and q for our model.

In this paper, a selection of p = 2 and q = 1 is made based on all the experimental datasets.

(Step 3): Using the prediction error series ε(k) to fit the GARCH model. This involves determining the values of p and q and estimating the corresponding parameters. However, it is difficult to determine the order of p and q either in theory or in practice. So, typically p = 1 and q = 1 is chosen as the standard order.

(Step 4): Verifying the model to determine if it is sufficient and accurate enough for making predictions. Typically, the Ljung–Box test is implemented for testing whether an ARCH effect exists in the normalized error of the fitted model. The fitting process may be repeated until there is no significant heteroscedasticity in the normalized error series. If the normalized error series are without heteroscedasticity, the model can be used for prediction.

(Step 5): Implementing the model to obtain one-step-ahead prediction. Once an appropriate ARIMA–GARCH model is constructed, the difference between range measurement and prediction $\left|\hat{d}(k)-d(k)\right|$ is used to detect the NLOS condition. θ is the NLOS detection threshold, which is determined by the GARCH model. If $\left|\hat{d}(k)-d(k)\right|>\theta$ , the sudden jump in the range estimate at the transition from LOS to NLOS usually detects the NLOS condition immediately. Then, the biased measurement from NLOS anchors is corrected by the range prediction $\hat{d}(k)$ .

## 4. The Global Fusion Strategy Aided by UWB

In order to enhance the positioning performance, GNSS, INS and other in-vehicle sensors are utilized to realize the global fusion. As previously mentioned, automobile motion can hardly be represented by one single model. Hence, it is more appropriate to employ multiple models to describe diverse vehicle motion. In this section, the adaptive IMM algorithm is developed to carry out the global fusion.

In the actual fulfillment of AIMM, the tightly coupled EKF algorithm is introduced to feed the GNSS measurement (L1, L2 pseudo-range) and UWB measurement (UWB range) to INS through an EKF filter even during GNSS partial outages (the number of visible GNSS satellites is less than four). Compared with the conventional loosely coupled EKF, the GNSS information can still be utilized if there are any visible GNSS satellites.

### 4.1. System Model Set

The kinematic vehicle models based on a reduced inertial sensor system (RISS), which integrates velocity provided by wheel speed sensors with the measurements from the two horizontal accelerometers and vertically aligned gyroscope, are employed to describe the system states.

In this paper, the constant turn (CT) and constant acceleration (CA) models are adopted to represent two typical vehicle motions, i.e., curvilinear and rectilinear movements, respectively. Both vehicle models have the same states, as follows:

(6) $$X\;=\;[\varphi \;\;\lambda \;\;h\;\;v^{e}\;\;v^{n}\;\;v^{u}\;p\;\;r\;\;A\;sf^{od}\;b^{\omega }b^{GPS}\;d^{GPS}\;b^{UWB}\;]'$$

where $\left[\varphi \;\;\lambda \;\;h\;\right]$ are the latitude, longitude, and altitude of vehicle, respectively; $\left[\;v^{e}\;\;v^{n}\;\;v^{u}\right]$ are the velocity components along the east, north, and up directions, respectively; [p  r  A] are the pitch angle, roll angle, and azimuth angle, respectively. The scale factor error of wheel speed is $sf^{od}$ ; $b^{\omega }$ is the stochastic gyroscope drift; $sf^{od}$ and $b^{\omega }$ are generally modeled by the Gauss–Markov model; $b^{GPS}$ is the GNSS receiver clock offset; $d^{GPS}$ is the clock drift error; $b^{UWB}\;$ is the UWB bias error term, which is modeled as a random constant process.

The GNSS receiver clock bias and drift error can be modeled as:

(7) $$\left[\begin{array}{l} \delta b_{k}^{GPS}\\ \delta d_{k}^{GPS} \end{array}\right]=\left[\begin{array}{l} 1\;\Delta t\\ 0\;1 \end{array}\right]\left[\begin{array}{l} \delta b_{k-1}^{GPS}\\ \delta d_{k-1}^{GPS} \end{array}\right]+\left[\begin{array}{l} \sigma_{b}\Delta t\\ \sigma_{d}\Delta t \end{array}\right]w_{k-1}^{GPS}$$

The CA model equation is shown as follows:

(8) $$X_{k,k-1}^{1}=f(X_{k-1}^{1},U_{k}^{1})=\left[\begin{array}{c} \varphi_{k-1}+\frac{v_{k-1}^{n}\Delta t+\frac{1}{2}(f_{k}^{x}\sin A_{k-1}-f_{k}^{y}\cos A_{k-1})\Delta t^{2}}{R_{M}+h_{k-1}}\\ \lambda_{k-1}+\frac{v_{k-1}^{e}\Delta t+\frac{1}{2}(f_{k}^{x}\sin A_{k-1}+f_{k}^{y}\cos A_{k-1})\Delta t^{2}}{(R_{N}+h_{k-1})\cos\varphi_{k-1}}\\ h_{k-1}+v_{k-1}^{u}\Delta t\\ v_{k}^{od}\sin A_{k-1}\cos p_{k-1}\\ v_{k}^{od}\cos A_{k-1}\cos p_{k-1}\\ v_{k}^{od}\sin p_{k-1}\\ \sin^{-1}(\frac{f_{k}^{y}-a_{k}^{od}}{g})\\ -\sin^{-1}(\frac{f_{k}^{x}}{g\cos p_{k-1}})\\ A_{k-1}+[(\omega^{e}\sin\varphi_{k-1})\Delta t+\frac{v_{k-1}^{e}\tan\varphi_{k-1}}{R_{N}+h_{k-1}}\Delta t]\\ (1-\gamma_{w}\Delta t)\delta sf_{k-1}^{od}+\sqrt{2\gamma_{w}\sigma_{w}^{2}\Delta t}\\ (1-\beta_{z}\Delta t)\delta b_{k-1}^{\omega }+\sqrt{2\beta_{z}\sigma_{z}^{2}\Delta t}\\ \delta b_{k-1}^{GPS}+\delta d_{k-1}^{GPS}\Delta t\\ \delta d_{k-1}^{GPS}\\ \delta b_{k-1}^{UWB} \end{array}\right]$$

where $R_{M}$ , $R_{N}$ are the meridian radius and the normal radius of curvature of the Earth, respectively. Δt is the sampling time. g is the acceleration of gravity. $\gamma_{w}$ , $\beta_{z}$ are the reciprocal of the autocorrelation time for the scale factor of wheel speed and the gyroscope’s stochastic drift, respectively. $\sigma_{w}$ , $\sigma_{z}$ are the variance of the noise associated with wheel speed and the gyroscope’s stochastic drift, respectively.

The measurements obtained from the accelerometers and wheel speed sensors comprise the input vector of CA model:

(9) $$U_{k}^{1}=\;[v^{od}\;a^{od}\;f^{x}\;f^{y}\;]'$$

where $v^{od}$ is the vehicle speed derived from the wheel speed sensors; $a^{od}$ is the vehicle acceleration derived from the wheel speed sensors; $f^{y}$ denotes the transversal acceleration, and $f^{x}$ denotes the forward acceleration acquired from the accelerometers, respectively.

The CT model equation is shown as follows:

(10) $$X_{k,k-1}^{2}=f(X_{k-1}^{2},U_{k}^{2})=\left[\begin{array}{c} \varphi_{k-1}+\frac{\left[v_{k-1}^{n}\frac{\sin\omega_{k}^{z}\Delta t}{\omega_{k}^{z}}+v_{k-1}^{e}\frac{\left(1-\cos\omega_{k}^{z}\Delta t\right)}{\omega_{k}^{z}}\right]}{R_{M}+h_{k-1}}\\ \lambda_{k-1}+\frac{\left[v_{k-1}^{e}\frac{\sin\omega_{k}^{z}\Delta t}{\omega_{k}^{z}}+v_{k-1}^{n}\frac{\left(\cos\omega_{k}^{z}\Delta t-1\right)}{\omega_{k}^{z}}\right]}{(R_{N}+h_{k-1})\cos\varphi_{k-1}}\\ h_{k-1}+v_{k-1}^{u}\Delta t\\ v_{k}^{od}\sin(A_{k-1}+\omega_{k}^{z}\Delta t)\cos p_{k-1}\\ v_{k}^{od}\cos(A_{k-1}+\omega_{k}^{z}\Delta t)\cos p_{k-1}\\ v_{k-1}^{u}\sin p_{k-1}\\ \sin^{-1}(\frac{f_{k}^{y}-a_{k}^{od}}{g})\\ -\sin^{-1}(\frac{f_{k}^{x}+v_{k}^{od}\omega_{k}^{z}}{g\cos p_{k-1}})\\ A_{k-1}+\omega_{k}^{z}\Delta t+[(\omega^{e}\sin\varphi_{k-1})\Delta t+\frac{v_{k-1}^{e}\tan\varphi_{k-1}}{R_{N}+h_{k-1}}\Delta t]\\ (1-\gamma_{w}\Delta t)\delta sf_{k-1}^{od}+\sqrt{2\gamma_{w}\sigma_{w}^{2}\Delta t}\\ (1-\beta_{z}\Delta t)\delta b_{k-1}^{\omega }+\sqrt{2\beta_{z}\sigma_{z}^{2}\Delta t}\\ \delta b_{k-1}^{GPS}+\delta d_{k-1}^{GPS}\Delta t\\ \delta d_{k-1}^{GPS}\\ \delta b_{k-1}^{UWB} \end{array}\right]$$

The measurements obtained from the wheel speed sensors and the yaw gyro comprise the input vector of the CT model:

(11) $$U_{k}^{2}=\;[v^{od}\;a^{od}\;\omega^{z}\;]'$$

where $\omega^{z}$ is the angular rate obtained from the vertically aligned gyroscope.

### 4.2. Observation Model

The observation information comes from UWB anchors and the GNSS. The measurement vector Z is:

(12) $$Z=\;[\;\rho^{GPS,1},\cdots ,\rho^{GPS,M},\;\;d^{UWB,1},\cdots ,d^{UWB,N}]'$$

where *M* is the number of available GNSSs, *N* is the number of UWB anchors. $\rho^{GPS,1},\cdots ,\rho^{GPS,M}$ are pseudo-range measurements of available GNSSs. $d^{UWB,1},\cdots ,d^{UWB,N}$ are the UWB range acquired from both LOS and NLOS anchors.

The quality of UWB’s observation can be significantly improved by the ARIMA–GARCH algorithm, which is given in Section 3. After the NLOS mitigation, the measurement vector can be rewritten as Equation (13):

(13) $$Z=\;[\;\rho^{GPS,1}\cdots \rho^{GPS,M},\;\;{\hat{d}}^{\mathrm{UWB},1}\cdots {\hat{d}}^{\mathrm{UWB},N}]'$$

The observation equation can be established as:

(14) $$Z_{k}=h(X_{k})=\;\left[\begin{array}{c} \sqrt{\begin{array}{c} {\left((R_{N}+h_{k})\cos\varphi_{k}\cos\lambda_{k}-x_{k}^{GPS,1}\right)}^{2}+{\left((R_{N}+h_{k})\cos\varphi_{k}\sin\lambda_{k}-y_{k}^{GPS,1}\right)}^{2}\\ +{\left((R_{N}(1-e^{2})+h_{k})\sin\varphi_{k}-z_{k}^{GPS,1}\right)}^{2} \end{array}}+\delta b_{k}^{GPS}\\ \vdots \\ \sqrt{\begin{array}{l} {\left((R_{N}+h_{k})\cos\varphi_{k}\cos\lambda_{k}-x_{k}^{GPS,M}\right)}^{2}+{\left((R_{N}+h_{k})\cos\varphi_{k}\sin\lambda_{k}-y_{k}^{GPS,M}\right)}^{2}\\ +{\left((R_{N}(1-e^{2})+h_{k})\sin\varphi_{k}-z_{k}^{GPS,M}\right)}^{2} \end{array}}+\delta b_{k}^{GPS}\\ \sqrt{\begin{array}{l} {\left((R_{N}+h_{k})\cos\varphi_{k}\cos\lambda_{k}-x_{k}^{UWB,1}\right)}^{2}+{\left((R_{N}+h_{k})\cos\varphi_{k}\sin\lambda_{k}-y_{k}^{UWB,1}\right)}^{2}\\ +{\left((R_{N}(1-e^{2})+h_{k})\sin\varphi_{k}-z_{k}^{UWB,1}\right)}^{2} \end{array}}+\delta b_{k}^{UWB}\\ \vdots \\ \sqrt{\begin{array}{l} {\left((R_{N}+h_{k})\cos\varphi_{k}\cos\lambda_{k}-x_{k}^{UWB,N}\right)}^{2}+{\left((R_{N}+h_{k})\cos\varphi_{k}\sin\lambda_{k}-y_{k}^{UWB,N}\right)}^{2}\\ +{\left((R_{N}(1-e^{2})+h_{k})\sin\varphi_{k}-z_{k}^{UWB,N}\right)}^{2} \end{array}}+\delta b_{k}^{UWB} \end{array}\right]$$

where $\left[x_{k}^{GPS,1}\;y_{k}^{GPS,1}\;z_{k}^{GPS,1}]\ldots [x_{k}^{GPS,M}\;y_{k}^{GPS,M}\;z_{k}^{GPS,M}\right]$ are the ECEF coordinates of GNSS satellites. $\left[x_{k}^{UWB,1}\;y_{k}^{UWB,1}\;z_{k}^{UWB,1}]\ldots [x_{k}^{UWB,N}\;y_{k}^{UWB,N}\;z_{k}^{UWB,N}\right]$ are the ECEF coordinates of UWB anchors.

### 4.3. Adaptive Algorithm of Markovian Probabilities Transition Matrix

In the traditional IMM approach, the individual probabilities of each model are derived from the incorporated measurements and the estimation itself, this approach makes the IMM completely self-contained, which means that no additional information is necessary for the estimation. However, there are many situations in which additional knowledge is available.

**Figure 5:**
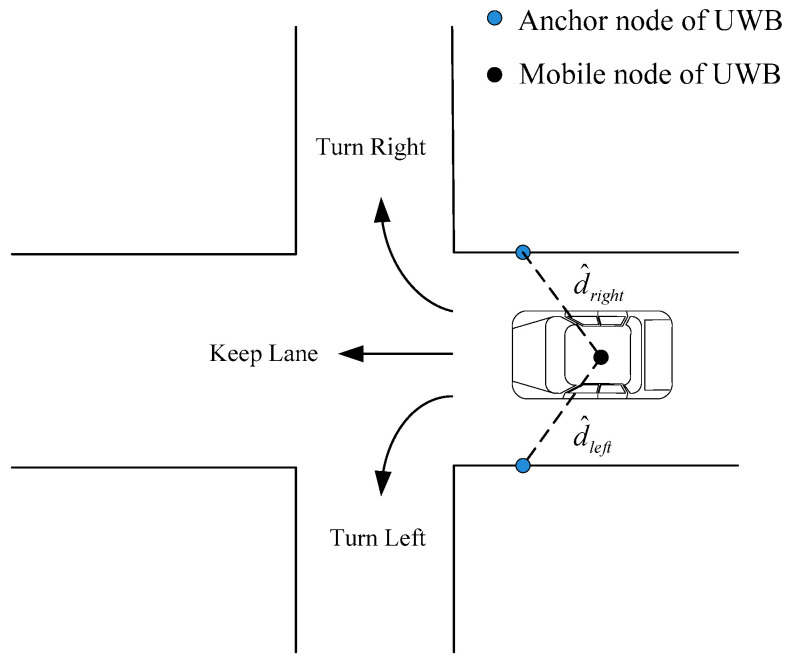
Three typical maneuvering scenarios at the intersection.

As is shown in Figure 5, if the vehicle drives straightly along the current lane, the distance between roadside UWB anchors (deployed at the left and right sides of the current lane) and the in-vehicle mobile node, ${\hat{d}}_{left}$ , ${\hat{d}}_{right}$ are approximately equal. Here, the distance difference is $\varepsilon_{uwb}$ ( $\varepsilon_{uwb}={\hat{d}}_{left}-{\hat{d}}_{right}$ ), and δ is the angle from the steering sensor.

When the vehicle turns left or right, $\varepsilon_{uwb}$ may decrease or increase apparently. The driving pattern of the vehicle can be identified preliminarily based on the information acquired from the roadside UWB nodes and the steering sensor. Generally, when the steering angle δ is quite small and the $\varepsilon_{uwb}$ approximately equals zero, the CA model is more suitable under this circumstance, and then the transition probabilities from the CA to CT model should be low. On the contrary, when the steering angle δ is large and the $\varepsilon_{uwb}$ decreases or increases apparently, this indicates that the vehicle may be in a highly curvilinear maneuvering, and thus the CT model is more suitable.

An adaptive algorithm based on Bayesian inference, which incorporates additional UWB and steering sensor information into the multiple-model estimation process, is developed to model the transition probabilities of the Markovian matrix adaptively, as follows:

(Step 1): Define the conditional probability tables (CPTs) of the Bayesian network under three different driving conditions (as shown in Figure 5 based on prior knowledge). For any particular assignment to the random variables of the network, full joint distributions may be calculated. That is, given that the number of random variables (denoted by $E_{i}$ ) in the network is *N*, the joint probability that they are assigned to a certain value $e_{i}$ is given by:

(15) $$P(e_{1},\ldots ,e_{i},\ldots ,e_{N})=\prod_{i=1}^{N}P(e_{i}{|\mathrm{Pa}(E}_{i}))$$

where ${\mathrm{Pa}(E}_{i})$ represents the set of parent nodes of $E_{i}$ .

(Step 2): Enter the observation $\varepsilon_{uwb}$ and δ as posterior evidence of time step *k*. And then, perform probabilistic reasoning within the Bayesian inference using the conditional probability Equation (15) and derive $\prod_{ij|k}$ .

(Step 3): Using $\prod_{ij|k}$ as the Markovian transition matrix of the AIMM method.

### 4.4. Global Fusion Algorithm Based on AIMM

To represent three typical vehicle maneuvers (keep lane, turn right, and turn left), the constant acceleration (CA) and the constant turn (CT) models are adopted, respectively. 

**Figure 6:**
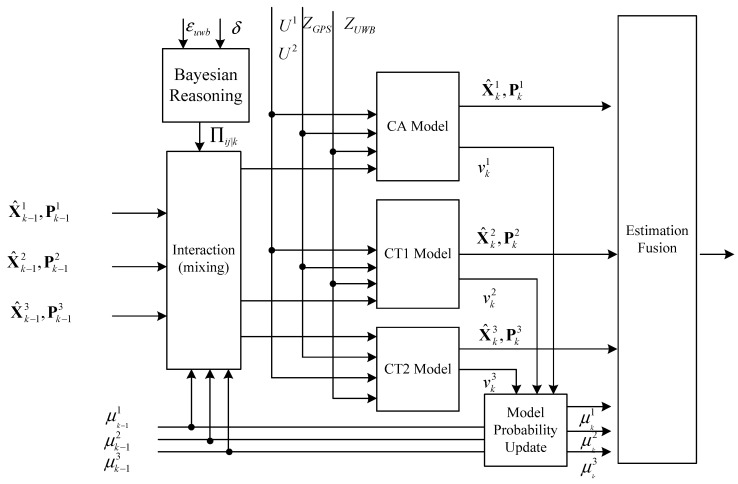
The AIMM algorithm.

Based on these models mentioned above, the AIMM algorithm mainly consists of four steps, as shown in Figure 6:

(1) Interaction:

Using the predicted model probability $\mu_{k-1}^{i}$ and the Markov transition probability $\pi_{ij}$ from the Markovian transition matrix $\Pi_{ij|k}$ , the individual filter estimate ${\hat{X}}_{k-1}^{i}$ of the *i-th* vehicle model (*i* = 1,2,3) is mixed with each other:

(16) $$\mu_{k,k-1}^{i}=\sum_{j=1}^{3}\pi_{ij}\mu_{k-1}^{j}\;(i=1,2,3)$$

The mixing weight is given by:

(17) μk−1j|i=πijμk−1jμk,k−1i (i,j=1,2,3)

The mixing of the state estimates ${\bar{X}}_{k-1}^{i}$ can be computed as:

(18) $${\bar{X}}_{k-1}^{i}=\sum_{j=1}^{3}\mu_{k-1}^{j|i}{\hat{X}}_{k-1}^{j}\;(i=1,2,3)$$

The mixing of the covariance ${\bar{P}}_{k-1}^{i}$ can be computed as:

(19) $${\bar{P}}_{k-1}^{i}=\sum_{j=1}^{3}\mu_{k-1}^{j|li}\left\{P_{k-1}^{j}+[{\bar{X}}_{k-1}^{i}-{\bar{X}}_{k-1}^{j}]{\left[{\bar{X}}_{k-1}^{i}-{\bar{X}}_{k-1}^{j}\right]}^{'}\right\}(i=1,2,3)$$

(2) Model individual filtering based on EKF:

Each filter utilizes its corresponding tightly coupled Extended Kalman Filter (EKF) model to predict and update its state and covariance. The execution of the *i-th* EKF (*i* = 1,2,3) can be described as follows:

(20) $${\hat{X}}_{k,k-1}^{i}=f_{i}({\bar{X}}_{k-1}^{i},U_{k}^{i})\;(i=1,2,3)$$

(21) $$g_{k}^{i}=Z_{k}-h({\hat{X}}_{k,k-1}^{i})\;(i=1,2,3)$$

(22) $$P_{k,k-1}^{i}=A_{k,k-1}^{i}{\bar{P}}_{k-1}^{i}A_{k,k-1}^{'i}+B_{k,k-1}^{i}\Gamma_{k-1}^{i}B_{k,k-1}^{'i}+Q_{k}^{i}\;(i=1,2,3)$$

(23) $$K_{k}^{i}=P_{k,k-1}^{i}H_{k}^{'}\cdot {\left[H_{k}P_{k,k-1}^{i}H_{k}^{'}+R_{k}\right]}^{-1}\;(i=1,2,3)$$

(24) $${\hat{X}}_{k}^{i}={\hat{X}}_{k,k-1}^{i}+K_{k}^{i}g_{k}^{i}\;(i=1,2,3)$$

(25) $$P_{k}^{i}=[1-K_{k}^{i}H_{k}]P_{k,k-1}^{i}\;(i=1,2,3)$$

where $A_{k,k-1}^{i}$ and $B_{k,k-1}^{i}$ are the Jacobian matrices of the process function $f^{i}(\cdot )$ with respect to ${\bar{X}}_{k-1}^{i}$ and $U_{k}^{i}$ . $P_{k,k-1}^{i}$ is the state prediction error covariance. $Q_{k}^{i}$ is the covariance matrix of the process noise. $\Gamma_{k-1}^{i}$ is the covariance matrix of the input noise. $K_{k}^{i}$ is the Kalman gain associated with the observation. $H_{k}$ is the Jacobian matrix of the observation function. $R_{k}$ is the measurement noise covariance. $P_{k}^{i}$ is the estimation error covariance.

(3) Model probability update:

According to the innovation error, each model probability is updated through Equation (26). Assuming Gaussian statistics, we derive the likelihood of the observation from the innovation vector $v_{k}^{i}$ and its covariance $s_{k}^{i}$ as follows:

(26) $$\Lambda_{k}^{i}=\frac{\exp\left\{-(1/2){\left(v_{k}^{i}\right)}^{'}(s_{k}^{i})v_{k}^{i}\right\}}{\sqrt{\left|2\pi s_{k}^{i}\right|}}(i=1,2,3)$$

where $v_{k}^{i}=g_{k}^{i}=Z_{k}-h({\hat{X}}_{k,k-1}^{i})\;$ , $s_{k}^{i}=P_{k,k-1}^{i}$ .

Then, the model probability update through Equation (27) is as follows:

(27) $$\mu_{k}^{i}=\frac{\mu_{k,k-1}^{i}\Lambda_{k}^{i}}{\sum_{j=1}^{3}\mu_{k,k-1}^{j}\Lambda_{k}^{j}}(i=1,2,3)$$

(4) Estimation fusion:

Finally, the combined state ${\hat{X}}_{k}$ and $P_{k}$ can be calculated as:

(28) $${\hat{X}}_{k}=\sum_{i=1}^{3}\mu_{k}^{i}{\hat{X}}_{k}^{i}$$

(29) $$P_{k}=\sum_{i=1}^{3}\mu_{k}^{i}\left\{P_{k}^{i}+[{\hat{X}}_{k}-{\hat{X}}_{k}^{i}]{\left[{\hat{X}}_{k}-{\hat{X}}_{k}^{i}\right]}^{'}\right\}$$

## 5. Experimental Results

**Figure 7:**
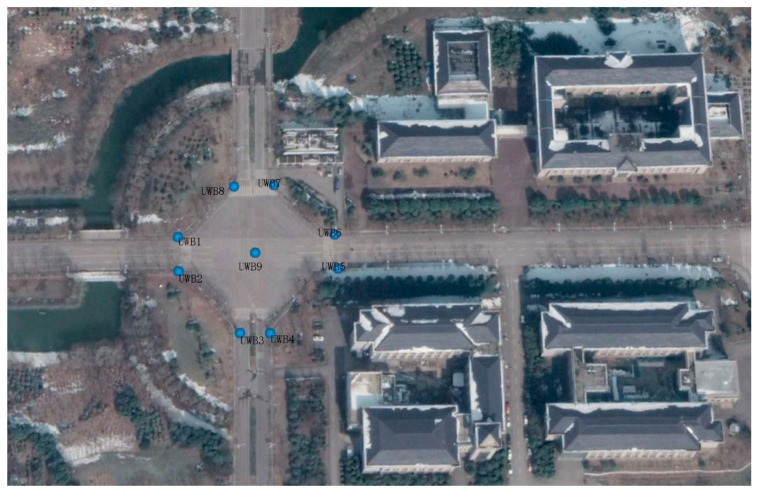
Bird’s-eye view of the intersection in ground test.

To evaluate the localization performance of the proposed solution, several experiments were conducted at an intersection in the Jiu Longhu campus of the southeast university, as is shown in Figure 7.

The vehicle used in our experiments was equipped with ABS and ESP, which allowed us to directly obtain information about the steering angle and wheel speed through the in-vehicle CAN bus. Additionally, the low-cost MEMS-based inertial sensors (VG440CA-200 IMU) sampled at 100 Hz and the consumer-grade GNSS receiver (NovAtel C260-AT) with a 1 Hz rate were equipped.

The RISS data utilized in this study were derived from the one vertical gyroscope and two horizontal accelerometers of the IMU (VG440CA-200); the gyroscope has a bias stability of 10°/h and angle random walk of $4.5^{o}/\sqrt{h}$ , while two accelerometers have a bias stability of 1 mg and velocity random walk of $1\;m/s/\sqrt{h}$ . The sensor accuracies (1σ) are 3 m and 0.05 m/s for the GNSS position and velocity, 0.05 m/s for wheel speed sensors, and 4° for the steering angle, respectively.

Typical driving maneuvering, such as lane keeping, left turn, right turn, ‘u’ turn, acceleration and deceleration, were conducted according to real driving scenarios. Using the setup described above, several ground test experiments were conducted along various trajectories.

### 5.1. The Deployment of UWB Anchors at the Intersection

**Figure 8:**
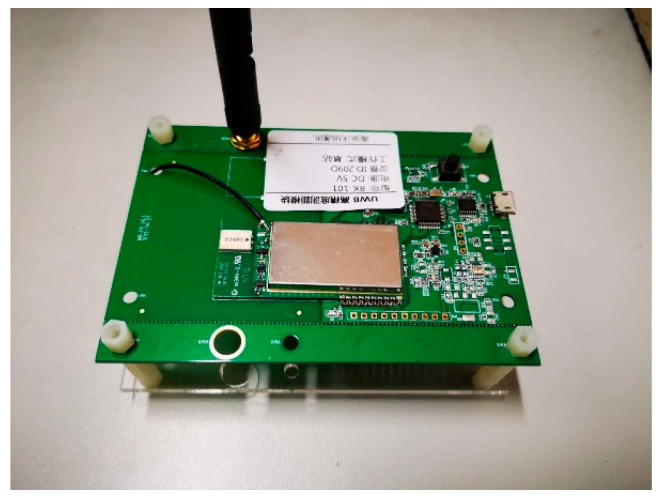
The RK-101 UWB module for the vehicle positioning experiment.

Low-cost UWB ranging modules (RK-101, Figure 8) based on 802.15.4a are employed in the vehicle positioning experiments. The max working distance of RK-101 is about 200 m. However, the range accuracy deteriorates when the distance between two UWB nodes is over 100 m. The measurement accuracy (1σ) is 0.5 m under LOS conditions.

UWB modules are deliberately deployed at the intersection, as shown in Figure 7. Each side of the four road segments is set an anchor node. Provided that there is a roundabout intersection, it will be quite easy to install a UWB node on road central infrastructures.

**Figure 9:**
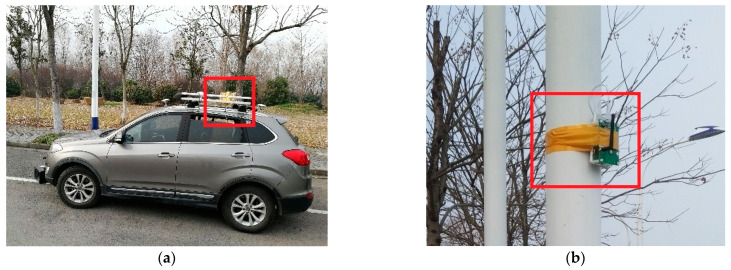
(**a**) Onboard mobile node, (**b**) Roadside anchor node.

Compared with roadside nodes (Figure 9), the central node has less probability of suffering from external interference or NLOS propagation. In view of this, one node is placed at the center of the road junction. 

This distribution of UWB anchors is capable of achieving a better geometric dilution of precision (GDOP) value at the intersection and leads to an accuracy improvement of UWB positioning.

Note that the number of UWB anchors depends on the needs of practical application. In our test, nine anchors in total cover the intersection areas (about $200\times 200\;m^{2}$ ). 

Taking cost cutting into account, the number of UWB anchors can be reduced (at least three anchors are needed to make sure that the UWB positioning system can work independently) at a smaller intersection.

### 5.2. Performance of UWB Preprocessing Algorithm

The ARIMA–GARCH algorithm’s performance, as discussed in Section 3, has been evaluated through repeated straight line and curve line ground tests. For brevity, only one test is presented here as an example; however, similar conclusions can be drawn from the other tests.

The residual weight least-squares (RWLS) method is introduced for multilateration [27]. All of the nine UWB anchors’ range measurements are adopted for the vehicle’s position calculation. Due to deliberate deployment of the anchor nodes, the vehicle can be located integrally when it drives passing by the crossroad.

**Figure 10:**
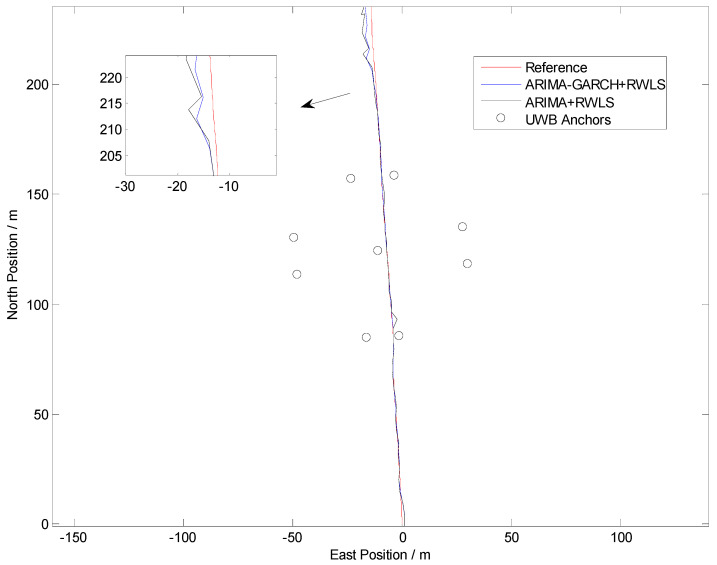
Positioning results in Trajectory I.

**Figure 11:**
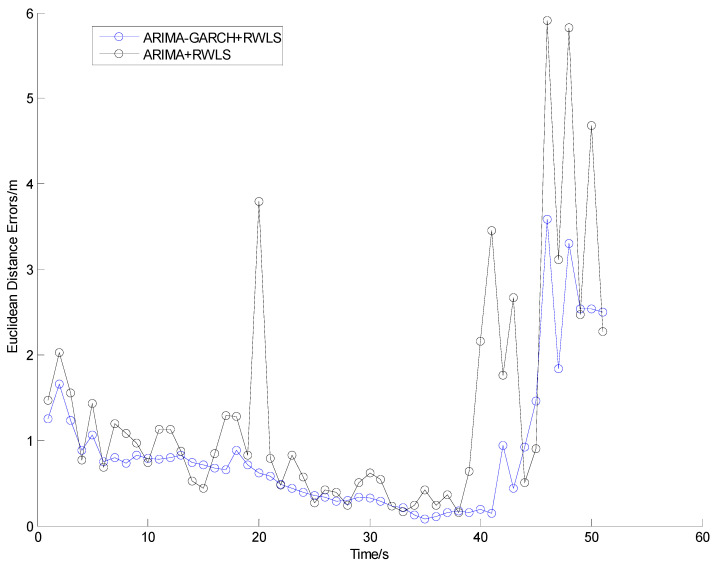
Euclidean distance errors in Trajectory I.

**Figure 12:**
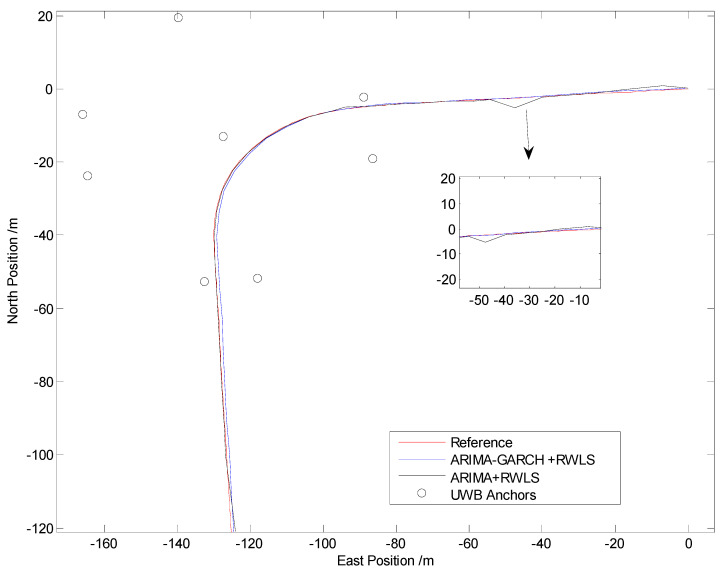
Positioning results in Trajectory II.

**Figure 13:**
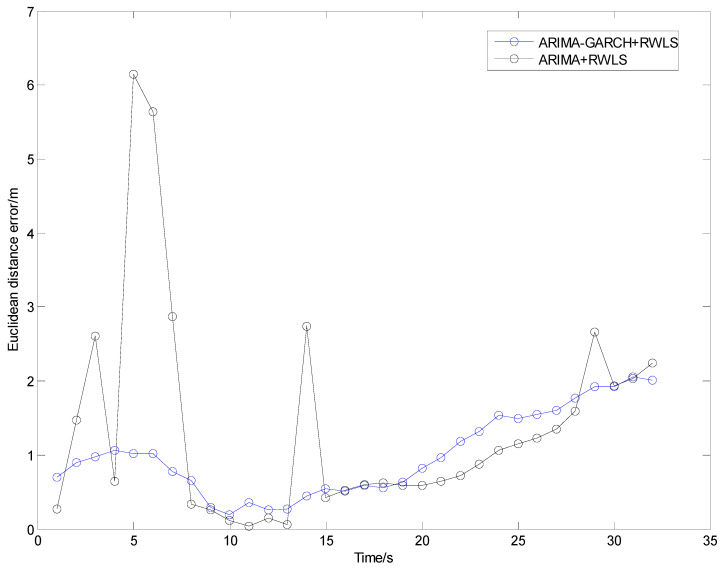
Euclidean distance errors in Trajectory II.

The positioning results of straight line and curve line tests are shown in Figure 10, Figure 11, Figure 12 and Figure 13, respectively.

From Figure 10 and Figure 12, it can be seen that some points of the black trajectories deviated from the references in red, especially at the start and the end of the route. When the vehicle drives into or drives away from the intersection, the positioning error becomes large. Since the vehicle is far from the center, frequent occurrences of NLOS propagation degrade the positioning performance.

Compared with the black ones, the blue trajectories are much more approximate to the references. It indicates that, after NLOS detection and mitigation by the ARIMA–GARCH algorithm, the positioning errors are reduced remarkably. Moreover, the ARIMA–GARCH method can achieve better performance than the ARIMA method, which is presented in [27].

Table 1 illustrates the statistics of positioning deviation (horizontal Euclidean distance), which include the maximum and root-mean-square (RMS) errors in Trajectory I and Trajectory II.

During the experiments, the parameters of the ARIMA model are p = 2, d = 1, q = 1, and the parameters of the GARCH model are p = 1, q = 1. As is shown in Table 1, when an appropriate ARIMA–GARCH model is built and utilized to mitigate abnormal errors in the range measurement of NLOS anchors, both the maximum error and the RMS error are reduced.

Note that 39% and 42% accuracy improvement values (in terms of RMS error) are achieved in tests I and II, respectively, which demonstrate that the ARIMA–GARCH method proposed in this paper is excellent for NLOS identification and correction of UWB in practical traffic scenarios.

### 5.3. Performance of the AIMM Algorithm

In order to validate performance of the AIMM fusion method proposed in Section 4, a further Test III is carried out.

Taking cost reducing into account, only two UWB anchors were used in Test III. Before the global fusion, raw measurements of the two UWB anchors are preprocessed by NLOS detection and mitigation algorithm based on the ARIMA–GARCH model, which is developed in Section 2.

The traditional single-model EKF and IMM method are investigated for performance comparison. Those three fusion methods can feed raw GNSS (L1 and L2 pseudo-range) and UWB measurements to MEMS-INS through EKF filters even when the number of visible satellites is three or fewer (GNSS partial outages), thereby verifying the performance improvement of the multisensor fusion method in degraded GNSS environments. In the whole test, only two satellites are used in the global fusion.

Before the actual implementation of AIMM fusion, we should define the conditional probability tables (CPTs) for the adaptive algorithm of the Markovian probabilities transition matrix. These conditional probabilities are mainly depended on prior knowledge, and we will be fine-tuning them through experimental data. $\varepsilon_{\mathrm{thr}}$ , $\delta_{\mathrm{thr}}$ are the threshold of distance difference and steer angle, respectively. The CPTs used in Test III are as shown in Table 2.

**Figure 14:**
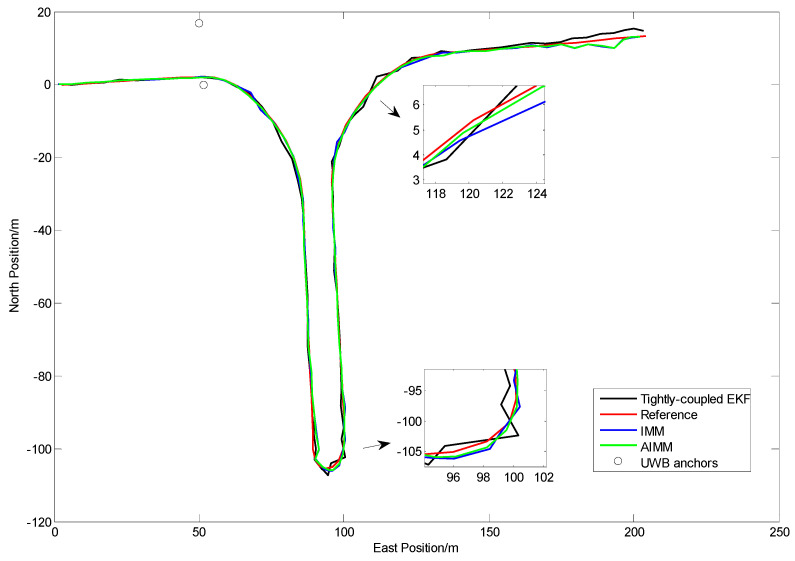
Positioning results in Trajectory III.

**Figure 15:**
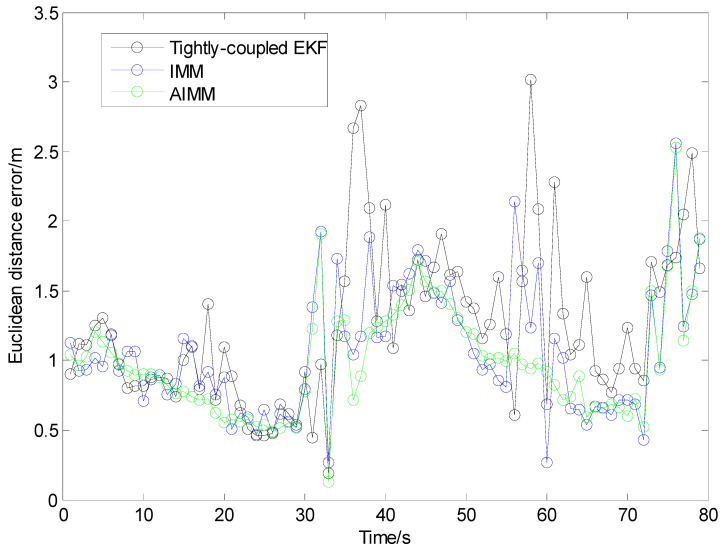
Euclidean distance errors in Trajectory III.

Figure 14 and Figure 15 and Table 3 show the positioning results in Test III.

From Figure 14, we can see that the trajectories of IMM and AIMM are more approximate to the reference, especially when the vehicle is changing lanes or turning around. As shown in Figure 15, the positioning error of the single-model EKF method starts to grow during 33~41 s (‘u’ turn) and 55~65 s (right turn), when the vehicle is in a highly curvilinear motion pattern. It indicates that the single-system model is not appropriate for tracking vehicles in frequent maneuvering, which is fairly common at intersections. The IMM and AIMM methods can achieve better performance under this circumstance. Owing to the adaptive algorithm of the Markovian probabilities transition matrix, the AIMM method performs better than the IMM, especially when there are frequent changes from rectilinear motion to curvilinear motion.

The statistics of horizontal positioning errors (Euclidean distance errors) in Trajectory III are shown in Table 3.

Owing to the NLOS mitigation algorithm and multi-sensor fusion strategy, all the three methods (even the tightly coupled EKF method) can still achieve lane-level positioning when the number of UWB anchors is cut to two.

Compared with traditional IMM methods, the proposed AIMM algorithm is capable of adjusting the model probabilities to make them more appropriate for current driving conditions, which then leads to the improvement of positioning performance. As is shown in Figure 15 and Table 3, the AIMM algorithm attains the best positioning accuracy among the three methods. In addition, the AIMM model could also assist in identifying the intention of the driver at intersections, which is quite useful to most vehicle active safety applications.

The AIMM algorithm provides a performance improvement of about 20% in terms of RMS error. As is shown in Table 3, the statistical error (RMS error) in Trajectory III of the AIMM method achieved 0.94 m. It can be attributed to the fact that this low-cost multi-sensor fusion approach is capable of achieving reliable and accurate localization at GNSS-challenging environments such as urban intersections.

## 6. Conclusions

UWB is recognized as a potential approach for positioning in GNSS-challenging environments. In this paper, a multi-sensor fusion strategy utilizing UWB, onboard sensors, and low-cost GNSS is proposed to achieve reliable and precise positioning at typical urban scenarios such as intersections. First, an ARIMA–GARCH model to address the NLOS problem of UWB at vehicular traffic scenarios is developed, and then the gross error in raw measurements of NLOS anchors can be corrected. Further, an algorithm based on Bayesian inference is developed to model the transition probabilities of the Markovian matrix adaptively, and then the AIMM algorithm is utilized to realize global fusion. For the proposed strategy, the effectiveness of both the UWB measurement preprocessing and the global fusion algorithms has been verified comprehensively. Experimental results demonstrate that this low-cost method is capable of achieving accurate, reliable, continuous, and integrated lane-level localization in GNSS-challenging environments.

## Figures and Tables

**Table 1:** Statistics of Euclidean distance errors in two trajectories (m).

Trajectory	ARIMA+RWLS		ARIMA–GARCH+RWLS	
	Max	RMS	Max	RMS
**I**	5.81	1.89	3.58	1.15
**II**	6.15	1.99	2.05	1.14

**Table 2:** Conditional probability tables of the adaptive algorithm.

**(1) Keep Lane**			
**Evidence $\varepsilon_{uwb}$ **	**Lane Change Left**	**Keep Lane**	**Lane Change Right**
$|\varepsilon_{uwb}|\leq \varepsilon_{\mathrm{thr}}$	0.005	0.99	0.005
$\varepsilon_{uwb}<-\varepsilon_{\mathrm{thr}}$	0.03	0.97	0.00
$\varepsilon_{uwb}>\varepsilon_{\mathrm{thr}}$	0.00	0.97	0.03
**Evidence δ **	**Lane Change Left**	**Keep Lane**	**Lane Change Right**
$|\delta |\leq \delta_{\mathrm{thr}}$	0.01	0.98	0.01
$\delta <-\delta_{\mathrm{thr}}$	0.03	0.96	0.01
$\delta >\delta_{\mathrm{thr}}$	0.01	0.96	0.03
**(2) Lane Change Left**			
**Evidence $\varepsilon_{uwb}$ **	**Lane Change Left**	**Keep Lane**	**Lane Change Right**
$|\varepsilon_{uwb}|\leq \varepsilon_{\mathrm{thr}}$	0.96	0.03	0.01
$\varepsilon_{uwb}<-\varepsilon_{\mathrm{thr}}$	0.98	0.02	0.00
$\varepsilon_{uwb}>\varepsilon_{\mathrm{thr}}$	0.96	0.01	0.03
**Evidence δ **	**Lane Change Left**	**Keep Lane**	**Lane Change Right**
$|\delta |\leq \delta_{\mathrm{thr}}$	0.96	0.03	0.01
$\delta <-\delta_{\mathrm{thr}}$	0.98	0.02	0.00
$\delta >\delta_{\mathrm{thr}}$	0.96	0.01	0.03
**(3) Lane Change Right**			
**Evidence $\varepsilon_{uwb}$ **	**Lane Change Left**	**Keep Lane**	**Lane Change Right**
$|\varepsilon_{uwb}|\leq \varepsilon_{\mathrm{thr}}$	0.01	0.03	0.96
$\varepsilon_{uwb}<-\varepsilon_{\mathrm{thr}}$	0.03	0.01	0.96
$\varepsilon_{uwb}>\varepsilon_{\mathrm{thr}}$	0.00	0.02	0.98
**Evidence δ **	**Lane Change Left**	**Keep Lane**	**Lane Change Right**
$|\delta |\leq \delta_{\mathrm{thr}}$	0.01	0.03	0.96
$\delta <-\delta_{\mathrm{thr}}$	0.03	0.01	0.96
$\delta >\delta_{\mathrm{thr}}$	0.00	0.02	0.98

**Table 3:** Statistics of Euclidean distance errors in Trajectory III (m).

Trajectory	Tightly Coupled EKF		IMM		AIMM	
	Max	RMS	Max	RMS	Max	RMS
III	3.2	1.37	2.6	1.19	2.6	0.94

## Data Availability

Data are contained within the article.
